# Supplementary material for: Oncogenes, tumor suppressor and differentiation genes represent the oldest human gene classes and evolve concurrently
Source: Sci Rep. 2019 Nov 11;9:16410. doi: 10.1038/s41598-019-52835-w (PMC6848199; doi:10.1038/s41598-019-52835-w)
Supplement: Supplementary file 1 — Supplementary Information [file 41598_2019_52835_MOESM1_ESM.pdf]

Oncogenes, tumor suppressor and differentiation genes represent the oldest human gene classes and evolve concurrently

A. Makashov[1,2,5], S.V. Malov[3,4] and A.P. Kozlov*[1,2,5,6]

[1]*Biomedical Center, Viborgskaya str. 8, Saint-Petersburg, Russia, 194044, Russia*

[2]*Peter the Great St. Petersburg Polytechnic University, Politekhnicheskaya ul., 29, St. Petersburg, 195251, Russia*

[3]*Theodosius Dobzhansky Center for Genome Bioinformatics, St.-Petersburg State University, 41A, Sredniy av., St. Petersburg, 199034, Russia*

[4]*Department of Mathematics, St.-Petersburg Electrotechnical University, 5, Prof. Popova str, St. Petersburg, 197376, Russia*

[5]*Research Institute of Ultra Pure Biologicals, 7 Pudozhskaya str., St. Petersburg, 197110, Russia*

[6]*Vavilov Institute of General Genetics, 3 Gubkina str., Moscow, 119333, Russia*

Author for Correspondence: A.P. Kozlov, contact@biomed.spb.ru

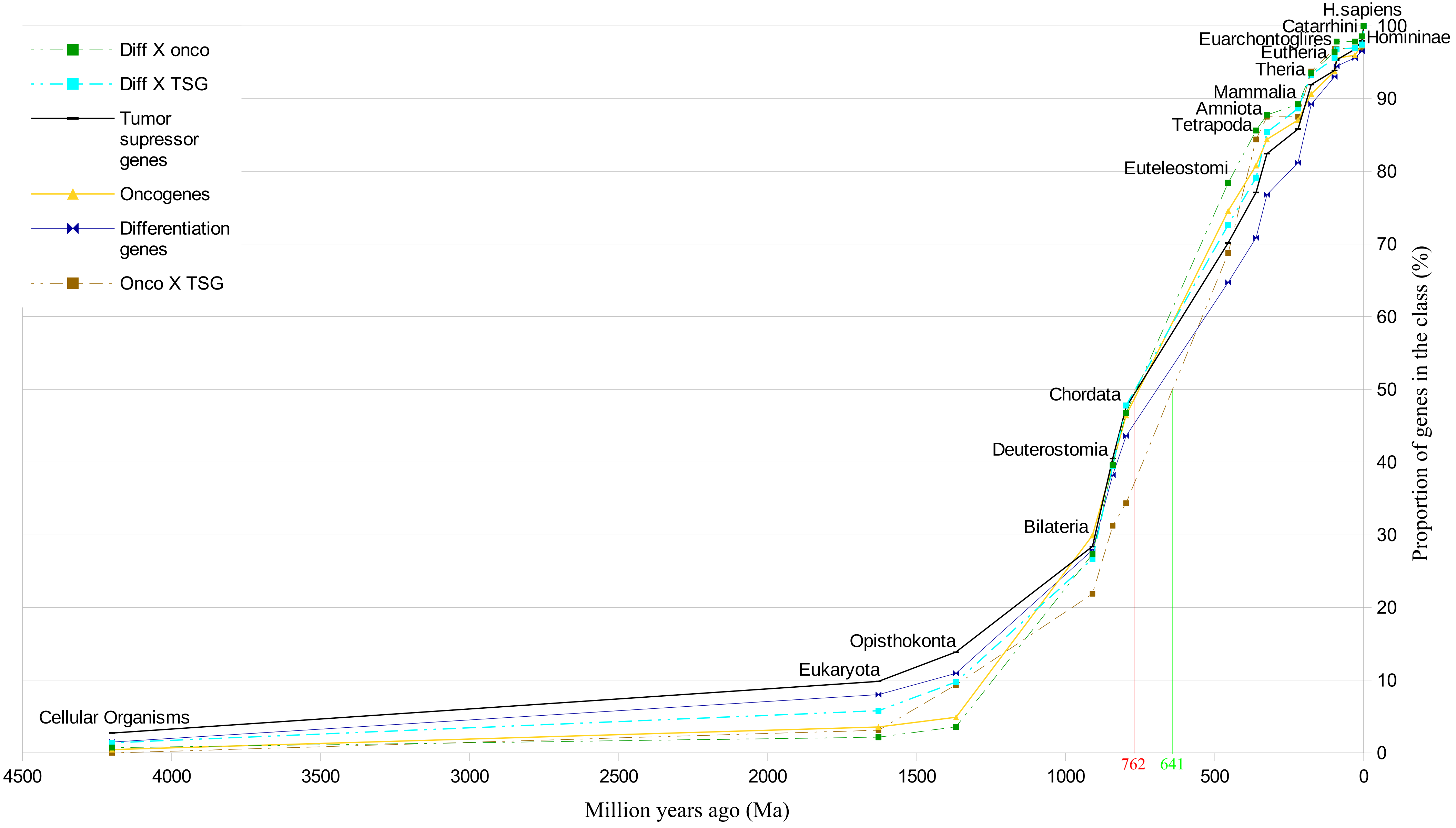

Supplementary Figure 1. Gene age distribution of pairwise overlapping subclasses of differentiation, onco-, and tumor suppressor genes.

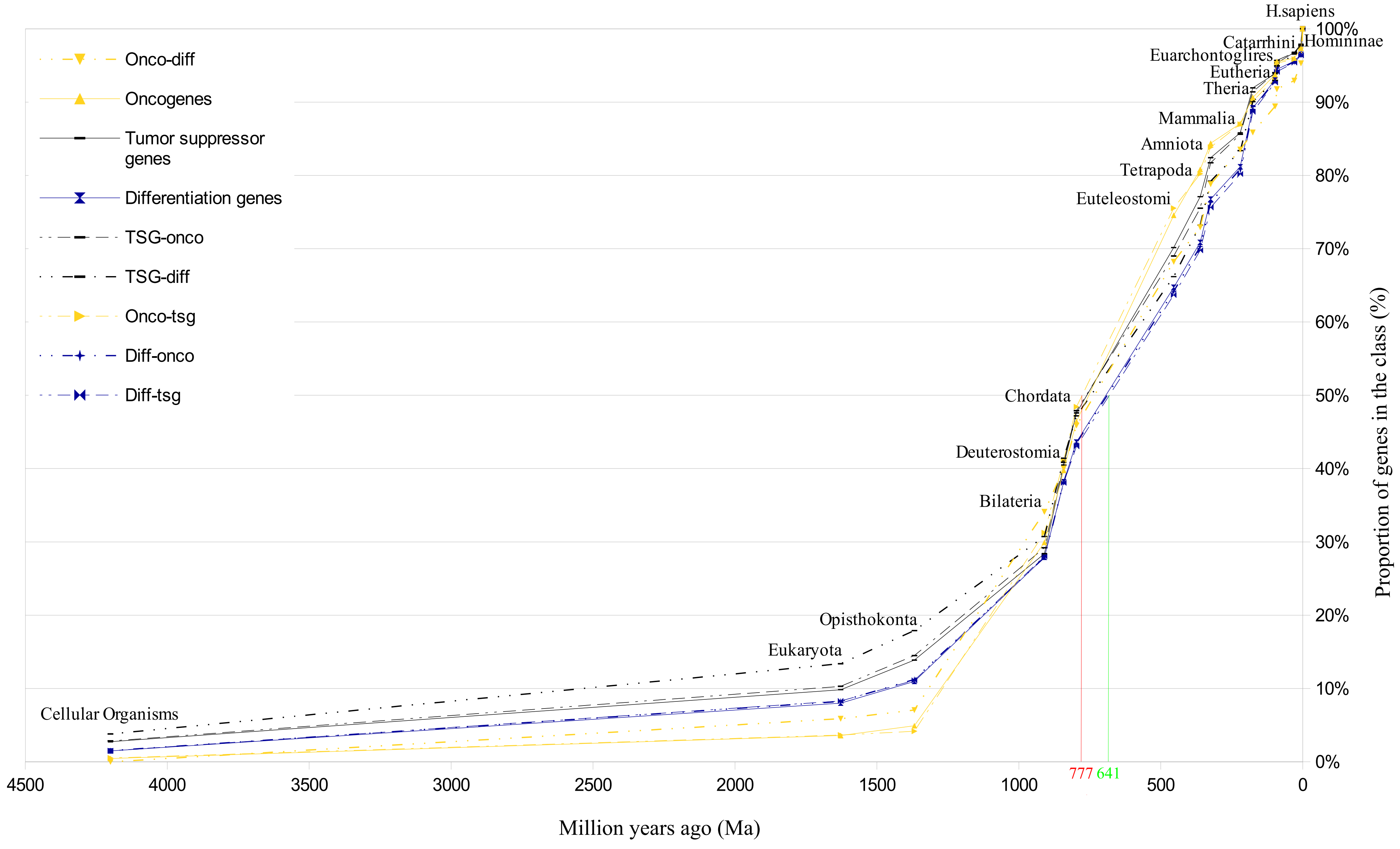

Supplementary Figure 2. Gene age distribution of pairwise subtraction subclasses of differentiation, onco-, and tumor suppressor genes

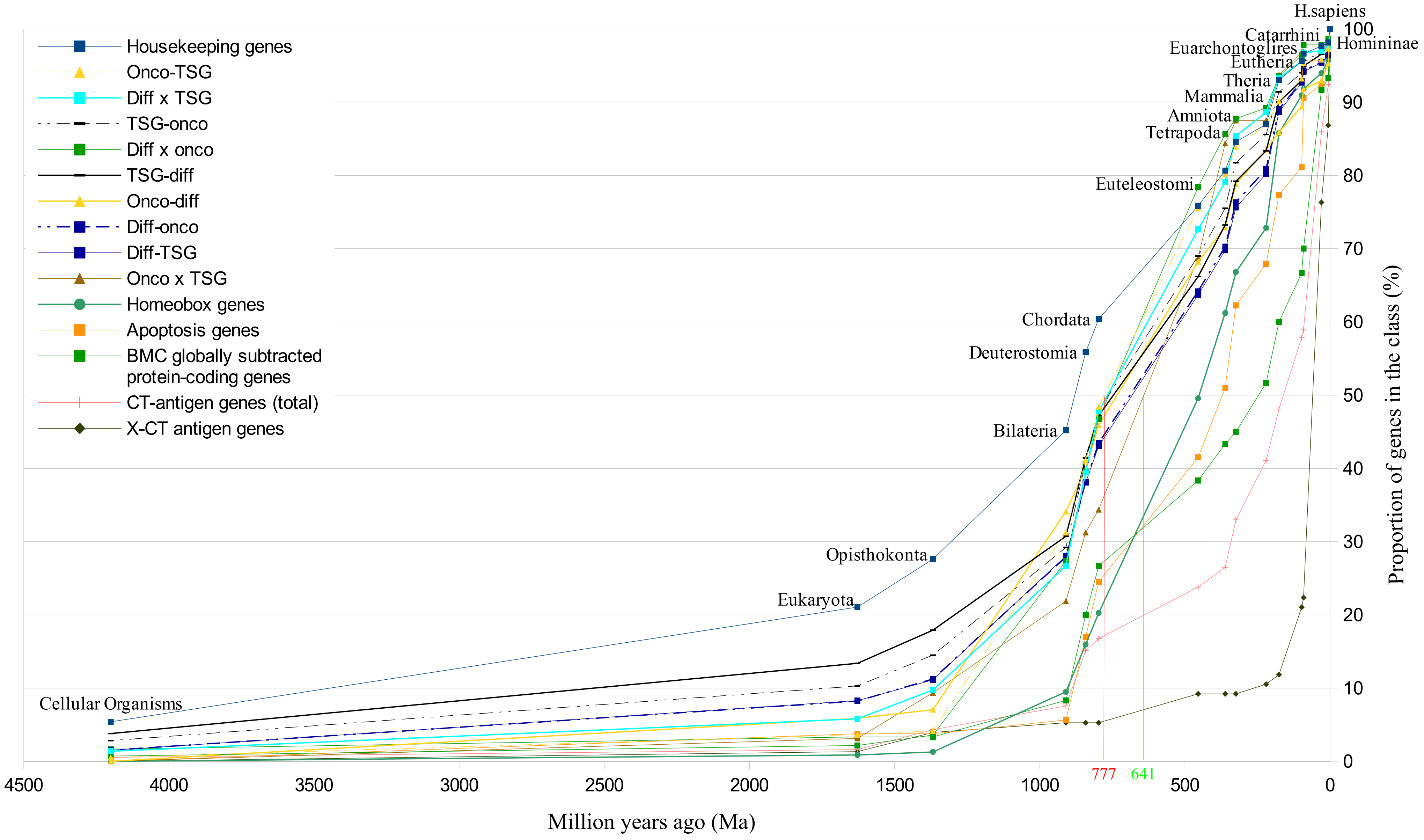

Supplementary Figure 3. Gene age distribution of pairwise overlapping and subtracted subclasses of differentiation, onco-, and tumor suppressor genes

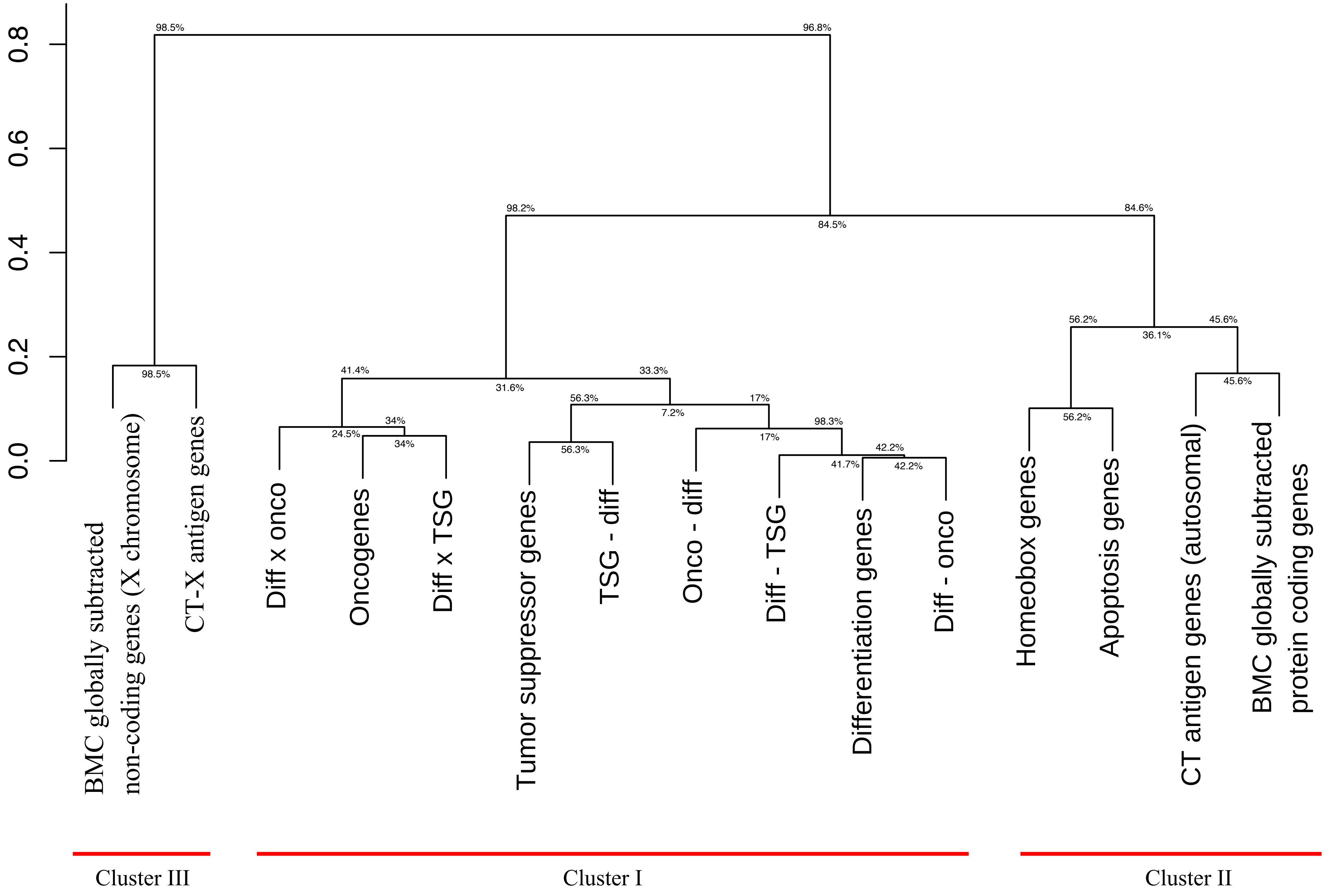

Supplementary Figure 4. Hierarchical classification of pairwise overlapping and subtracted subclasses of differentiation, onco-, and tumor suppressor genes (Kolmogorov-Smirnov distance, complete linkage)

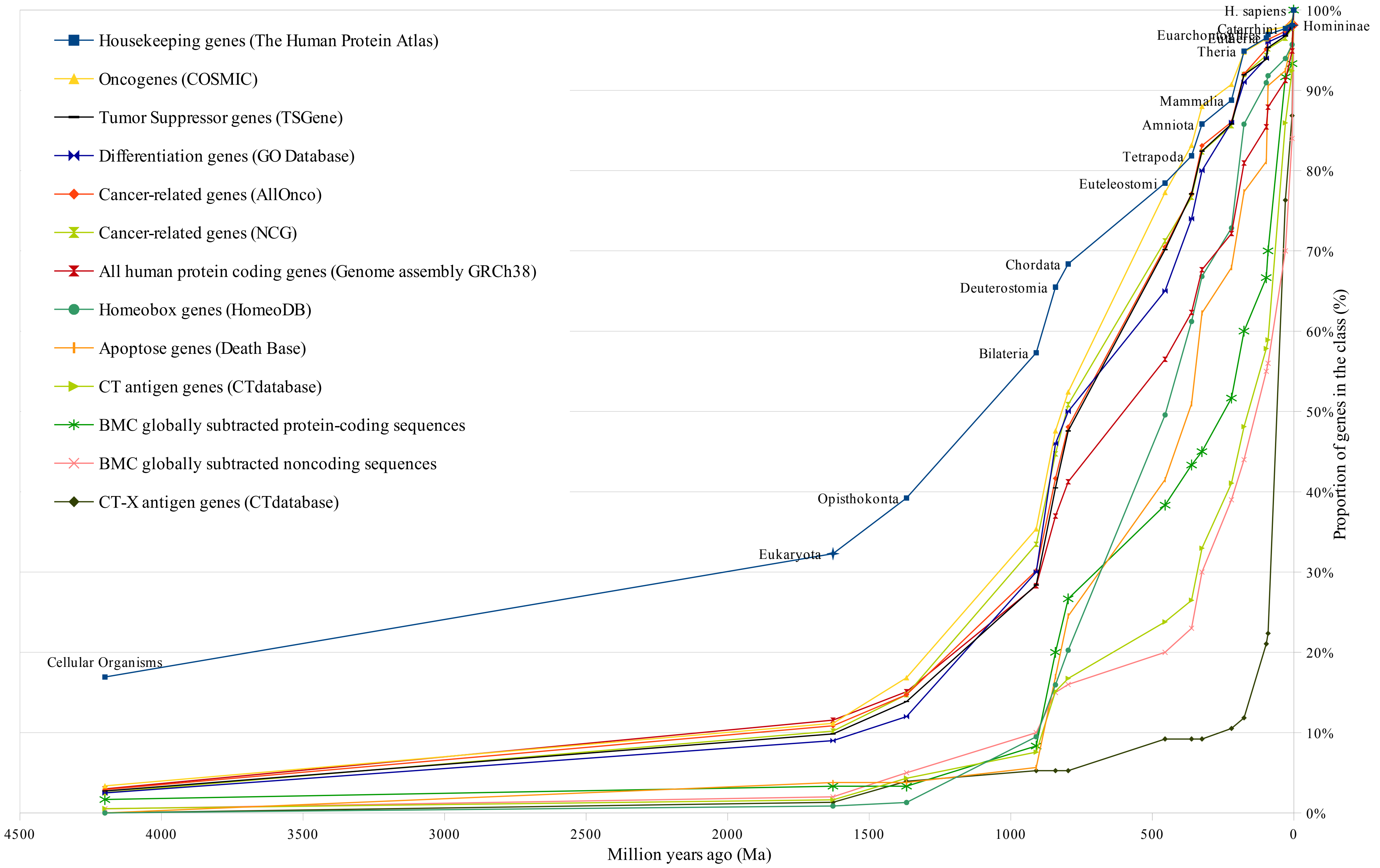

Supplementary Figure 5. One of previous versions of gene age distribution curves

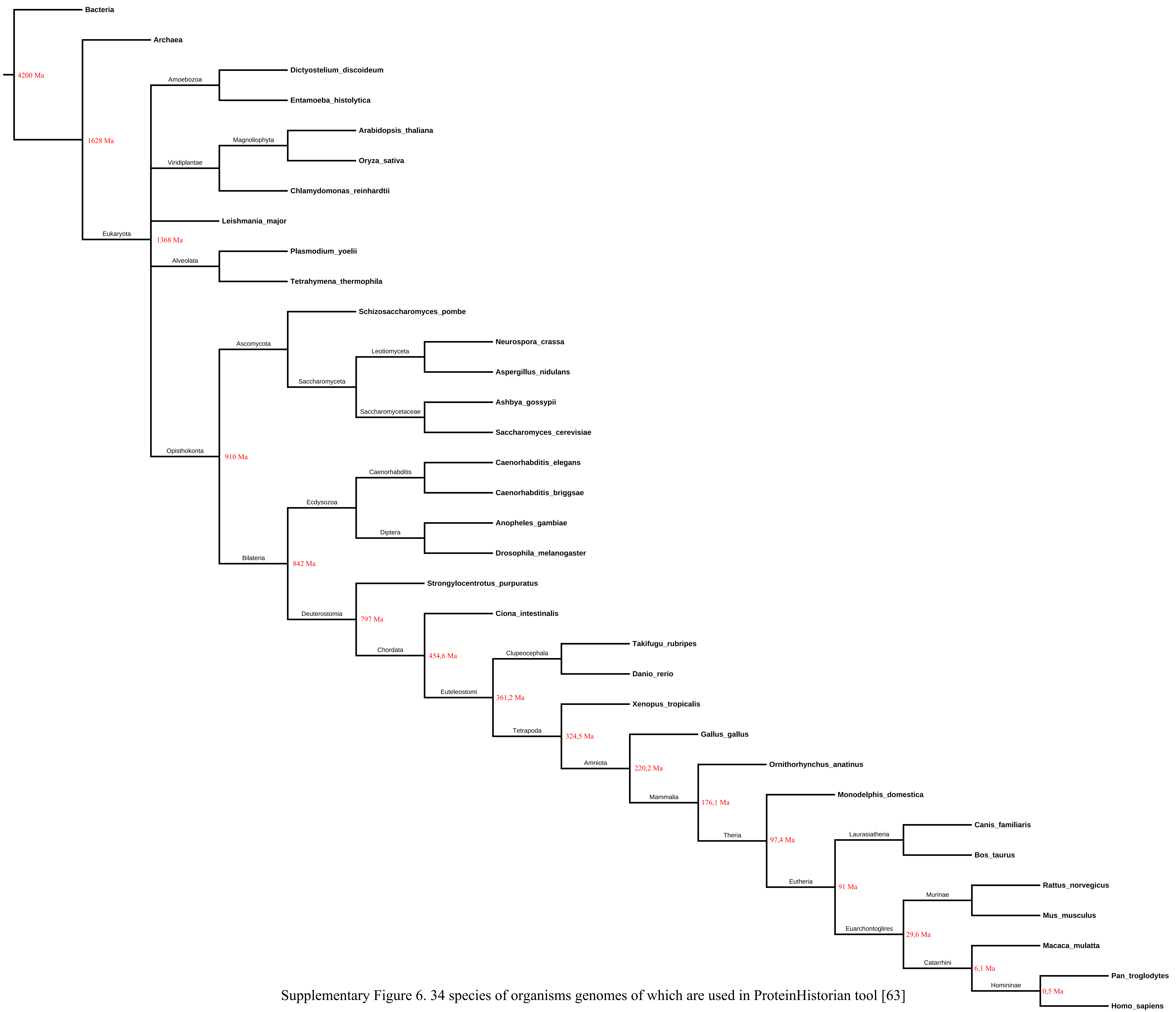

Supplementary Figure 6. 34 species of organisms genomes of which are used in ProteinHistorian tool [63]

Supplementary list 1. Genomes used for phylogenetic analysis of non-coding genes

1. Escherichia coli str. K-12 substr. MG1655
2. Sulfolobus islandicus L.S.2.15
3. Plasmodium yoelii (assembly PY17X01)
4. Saccharomyces cerevisiae S288C (assembly R64)
5. Caenorhabditis elegans (assembly WBcel235)
6. Drosophila melanogaster (assembly Release 6 plus ISO1 MT)
7. Strongylocentrotus purpuratus (assembly Spur_4.2)
8. Ciona intestinalis (assembly KH)
9. Takifugu rubripes (assembly FUGU5)
10. Danio rerio (assembly GRCz11)
11. Xenopus tropicalis (assembly Xenopus_tropicalis_v9.1)
12. Crocodylus porosus (assembly CroPor_comp1)
13. Gallus gallus (assembly Gallus_gallus-5.0)
14. Ornithorhynchus anatinus (assembly Ornithorhynchus_anatinus-5.0.1)
15. Monodelphis domestica (assembly MonDom5)
16. Canis lupus familiaris (assembly CanFam3.1)
17. Bos taurus (assembly Bos_taurus_UMD_3.1.1)
18. Ovis aries (assembly Oar_v4.0)
19. Mus musculus (assembly GRCm38.p6)
20. Rattus norvegicus (assembly Rnor_6.0)
21. Macaca mulatta (assembly Mmul_8.0.1)
22. Nomascus leucogenys (assembly Nleu_3.0)
23. Pongo abelii (assembly Susie_PABv2)
24. Gorilla gorilla gorilla (assembly gorGor4)
25. Pan troglodytes (assembly Clint_PTRv2)
